# 4-Hy­droxy-2-methyl-1,1-dioxo-*N*-phenyl-2*H*-1λ^6^,2-benzothia­zine-3-carboxamide

**DOI:** 10.1107/S1600536812021708

**Published:** 2012-05-19

**Authors:** Farhana Aman, Waseeq Ahmad Siddiqui, Adnan Ashraf, Hamid Latif Siddiqui, Masood Parvez

**Affiliations:** a Department of Chemistry, University of Sargodha, Sargodha 40100, Pakistan; b Institute of Chemistry, University of the Punjab, Lahore 54590, Pakistan; c Department of Chemistry, The University of Calgary, 2500 University Drive NW, Calgary, Alberta, Canada T2N 1N4

## Abstract

In the title mol­ecule, C_16_H_14_N_2_O_4_S, the thia­zine ring adopts a twist chair conformation with the N and adjacent C atom displaced by 0.966 (3) and 0.386 (4) Å, respectively, on the same side of the mean plane formed by the remaining ring atoms. The dihedral angle between the mean planes of the benzene rings is 37.65 (10)°. The mol­ecular structure features an intra­molecular O—H⋯O hydrogen bond, which generates an *S*(6) ring. In the crystal, mol­ecules are linked by N—H⋯O and C—H⋯O hydrogen bonds.

## Related literature

For background to the biological activity of benzothia­zine derivatives, and further synthetic details, see: Siddiqui *et al.* (2007 ▶); Ahmad *et al.* (2010 ▶). For related structures, see: Siddiqui *et al.* (2008 ▶; 2009 ▶).
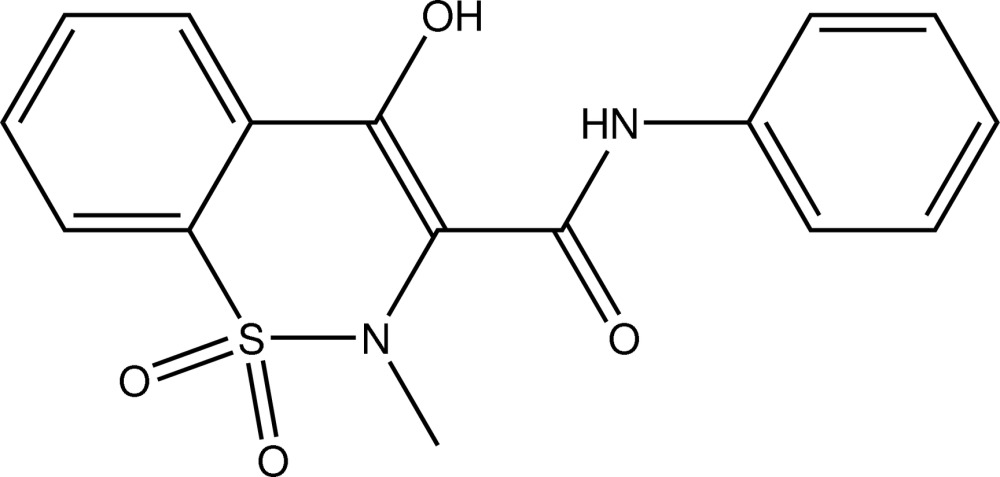


## Experimental

### 

#### Crystal data

C_16_H_14_N_2_O_4_S
*M* *_r_* = 330.35
Monoclinic, 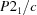

*a* = 10.502 (2) Å
*b* = 7.649 (3) Å
*c* = 19.235 (4) Å
β = 103.395 (15)°
*V* = 1503.1 (7) Å^3^
*Z* = 4
Mo *K*α radiation
μ = 0.24 mm^−1^
*T* = 173 K
0.10 × 0.08 × 0.07 mm

#### Data collection

Nonius KappaCCD diffractometer
Absorption correction: multi-scan (*SORTAV*; Blessing, 1997 ▶) *T*_min_ = 0.977, *T*_max_ = 0.984
12580 measured reflections
3450 independent reflections
2901 reflections with *I* > 2σ(*I*)
*R*_int_ = 0.059

#### Refinement

*R*[*F* ^2^ > 2σ(*F* ^2^)] = 0.048
*wR*(*F* ^2^) = 0.119
*S* = 1.06
3450 reflections
210 parameters
H-atom parameters constrained
Δρ_max_ = 0.52 e Å^−3^
Δρ_min_ = −0.31 e Å^−3^

### 

Data collection: *COLLECT* (Hooft, 1998 ▶); cell refinement: *DENZO* (Otwinowski & Minor, 1997 ▶); data reduction: *SCALEPACK* (Otwinowski & Minor, 1997 ▶); program(s) used to solve structure: *SHELXS97* (Sheldrick, 2008 ▶); program(s) used to refine structure: *SHELXL97* (Sheldrick, 2008 ▶); molecular graphics: *ORTEP-3* (Farrugia, 1997 ▶); software used to prepare material for publication: *SHELXL97*.

## Supplementary Material

Crystal structure: contains datablock(s) global, I. DOI: 10.1107/S1600536812021708/hb6789sup1.cif

Structure factors: contains datablock(s) I. DOI: 10.1107/S1600536812021708/hb6789Isup2.hkl

Supplementary material file. DOI: 10.1107/S1600536812021708/hb6789Isup3.cml

Additional supplementary materials:  crystallographic information; 3D view; checkCIF report

## Figures and Tables

**Table 1:** Hydrogen-bond geometry (Å, °)

*D*—H⋯*A*	*D*—H	H⋯*A*	*D*⋯*A*	*D*—H⋯*A*
N2—H2*N*⋯O1^i^	0.88	2.26	2.987 (2)	140
C3—H3⋯O2^ii^	0.95	2.48	3.300 (3)	145
C13—H13⋯O4^iii^	0.95	2.48	3.339 (3)	151
O3—H3*O*⋯O4	0.84	1.79	2.534 (2)	146

Symmetry codes: (i) 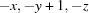; (ii) 
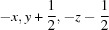
; (iii) 
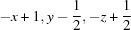
.

**Fig. 1.**
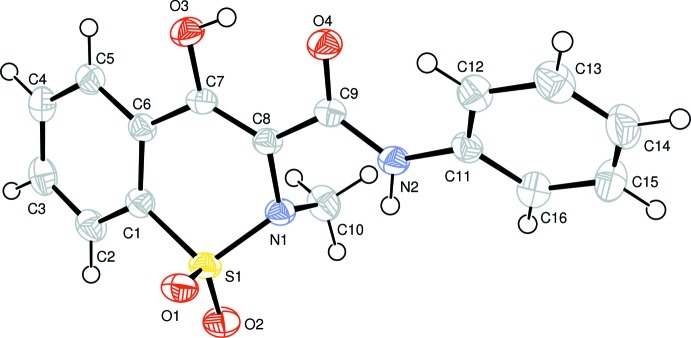
The molecular structure of the title compound with displacement ellipsoids drawn at the 50% probability level. H atoms are presented as small spheres of arbitrary radius.

**Fig. 2.**
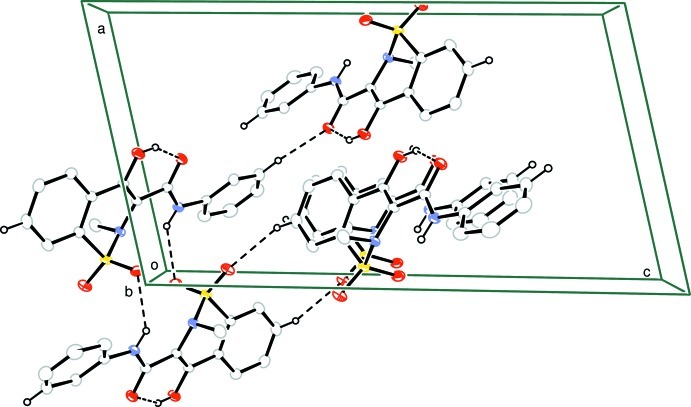
A part of the unit cell showing hydrogen bonding interactions (dotted lines) in the crystal structure of the title compound. H atoms non-participating in hydrogen-bonding were omitted for clarity.

## supplementary crystallographic information

### Comment

In continuation of our research on the synthesis of potentially biologically active 1,2-benzothiazine 1,1-dioxide derivatives (Siddiqui *et al.*, 2007; Ahmad *et al.*, 2010) herein, we report the synthesis and crystal structure of the title compound.

The bond distances and angles in the title compound (Fig. 1) agree very well with the corresponding bond distances and angles reported in closely related compounds (Siddiqui *et al.*, 2008; 2009). The heterocyclic thiazine ring adopts a twist chair conformation with atoms S1 and C1 displaced by 0.966 (3) and 0.386 (4) Å, respectively, on the same side from the mean plane formed by the remaining ring atoms (r.m.s. deviation 0.004 for N1/C6–C8 atoms). The mean-plane of the benzene rings C1–C6 and C11–C16 are inclined at a dihedral angle 37.65 (10)° with respect to each other.

The molecular structure is stabilized by an intra-molecular O3—H3O···O4 hydrogen bond. The crystal packing is consolidated by intermolecular N2—H2N···O1, C3—H3···O2 and C13—H13···O4 hydrogen bonds (Fig. 2 and Table 1).

### Experimental

A mixture of 4-hydroxy-2-methyl-2*H*-1,2 benzothiazine-3-carboxylic acid methyl ester 1,1 dioxide (0.65 g, 2.42 mmol) and aniline (0.65 ml, 2.42 mmol) in xylene (150 ml) was allowed to react as per reported procedure (Siddiqui *et al.*, 2007) to isolate the title compound. Colourless prisms were grown from ethyl acetate solution by slow evaporation at room temperature; m.p. = 484–485 K

### Refinement

All H atoms were positioned geometrically and refined using a riding model, with O—H = 0.84, N—H = 0.88 Å and C—H = 0.95 and 0.98 Å, for aryl and methyl H-atoms, respectively. The *U*_iso_(H) were allowed at 1.5*U*_eq_(O) or 1.2*U*_eq_(C/N).

### Crystal data

**Table:**

C_16_H_14_N_2_O_4_S	*F*(000) = 688
*M**_r_* = 330.35	*D*_x_ = 1.460 Mg m^−^^3^
Monoclinic, *P*2_1_/*c*	Mo *K*α radiation, λ = 0.71073 Å
Hall symbol: -P 2ybc	Cell parameters from 6808 reflections
*a* = 10.502 (2) Å	θ = 1.0–27.5°
*b* = 7.649 (3) Å	µ = 0.24 mm^−^^1^
*c* = 19.235 (4) Å	*T* = 173 K
β = 103.395 (15)°	Prism, colorless
*V* = 1503.1 (7) Å^3^	0.10 × 0.08 × 0.07 mm
*Z* = 4	

### Data collection

**Table:**

Nonius KappaCCD diffractometer	3450 independent reflections
Radiation source: fine-focus sealed tube	2901 reflections with *I* > 2σ(*I*)
Graphite monochromator	*R*_int_ = 0.059
ω and φ scans	θ_max_ = 27.6°, θ_min_ = 2.0°
Absorption correction: multi-scan (*SORTAV*; Blessing, 1997)	*h* = −13→13
*T*_min_ = 0.977, *T*_max_ = 0.984	*k* = −9→9
12580 measured reflections	*l* = −24→24

### Refinement

**Table:**

Refinement on *F*^2^	Primary atom site location: structure-invariant direct methods
Least-squares matrix: full	Secondary atom site location: difference Fourier map
*R*[*F*^2^ > 2σ(*F*^2^)] = 0.048	Hydrogen site location: difference Fourier map
*wR*(*F*^2^) = 0.119	H-atom parameters constrained
*S* = 1.06	*w* = 1/[σ^2^(*F*_o_^2^) + (0.0433*P*)^2^ + 1.2798*P*] where *P* = (*F*_o_^2^ + 2*F*_c_^2^)/3
3450 reflections	(Δ/σ)_max_ = 0.002
210 parameters	Δρ_max_ = 0.52 e Å^−^^3^
0 restraints	Δρ_min_ = −0.31 e Å^−^^3^

### Special details

**Table:**

Geometry. All e.s.d.'s (except the e.s.d. in the dihedral angle between two l.s. planes) are estimated using the full covariance matrix. The cell e.s.d.'s are taken into account individually in the estimation of e.s.d.'s in distances, angles and torsion angles; correlations between e.s.d.'s in cell parameters are only used when they are defined by crystal symmetry. An approximate (isotropic) treatment of cell e.s.d.'s is used for estimating e.s.d.'s involving l.s. planes.
Refinement. Refinement of *F*^2^ against ALL reflections. The weighted *R*-factor *wR* and goodness of fit *S* are based on *F*^2^, conventional *R*-factors *R* are based on *F*, with *F* set to zero for negative *F*^2^. The threshold expression of *F*^2^ > σ(*F*^2^) is used only for calculating *R*-factors(gt) *etc*. and is not relevant to the choice of reflections for refinement. *R*-factors based on *F*^2^ are statistically about twice as large as those based on *F*, and *R*- factors based on ALL data will be even larger.

### Fractional atomic coordinates and isotropic or equivalent isotropic displacement parameters (Å^2^)

**Table:**

	*x*	*y*	*z*	*U*_iso_*/*U*_eq_	
S1	0.06977 (5)	0.65050 (7)	−0.08374 (3)	0.02930 (15)	
O1	0.03451 (13)	0.7324 (2)	−0.02412 (8)	0.0343 (4)	
O2	−0.03040 (14)	0.5885 (2)	−0.14150 (8)	0.0397 (4)	
O3	0.46114 (13)	0.7361 (2)	0.03008 (8)	0.0336 (3)	
H3O	0.4835	0.6611	0.0625	0.050*	
O4	0.44020 (13)	0.4617 (2)	0.10046 (7)	0.0327 (3)	
N1	0.16698 (15)	0.4874 (2)	−0.05340 (8)	0.0269 (4)	
N2	0.25707 (16)	0.2921 (2)	0.06629 (9)	0.0286 (4)	
H2N	0.1836	0.2817	0.0335	0.034*	
C1	0.1745 (2)	0.7916 (3)	−0.11620 (10)	0.0290 (4)	
C2	0.1311 (2)	0.8897 (3)	−0.17712 (11)	0.0344 (5)	
H2	0.0429	0.8807	−0.2036	0.041*	
C3	0.2174 (2)	1.0016 (3)	−0.19931 (12)	0.0374 (5)	
H3	0.1892	1.0690	−0.2415	0.045*	
C4	0.3453 (2)	1.0147 (3)	−0.15962 (12)	0.0349 (5)	
H4	0.4040	1.0927	−0.1747	0.042*	
C5	0.3885 (2)	0.9167 (3)	−0.09875 (11)	0.0312 (4)	
H5	0.4762	0.9286	−0.0719	0.037*	
C6	0.30455 (19)	0.8003 (3)	−0.07627 (10)	0.0267 (4)	
C7	0.34974 (18)	0.6857 (3)	−0.01458 (10)	0.0265 (4)	
C8	0.28462 (18)	0.5399 (3)	−0.00344 (10)	0.0258 (4)	
C9	0.33296 (18)	0.4289 (3)	0.05864 (10)	0.0275 (4)	
C10	0.1852 (2)	0.3560 (3)	−0.10655 (12)	0.0386 (5)	
H10A	0.2385	0.2592	−0.0821	0.046*	
H10B	0.2296	0.4101	−0.1406	0.046*	
H10C	0.0997	0.3115	−0.1322	0.046*	
C11	0.28010 (19)	0.1627 (3)	0.12035 (11)	0.0293 (4)	
C12	0.3610 (2)	0.1906 (3)	0.18722 (11)	0.0338 (5)	
H12	0.4041	0.2997	0.1986	0.041*	
C13	0.3782 (2)	0.0577 (3)	0.23736 (13)	0.0426 (6)	
H13	0.4344	0.0758	0.2832	0.051*	
C14	0.3156 (3)	−0.0998 (4)	0.22204 (15)	0.0486 (6)	
H14	0.3279	−0.1898	0.2570	0.058*	
C15	0.2342 (3)	−0.1267 (3)	0.15529 (15)	0.0497 (6)	
H15	0.1904	−0.2355	0.1445	0.060*	
C16	0.2163 (2)	0.0035 (3)	0.10429 (13)	0.0395 (5)	
H16	0.1606	−0.0156	0.0584	0.047*	

### Atomic displacement parameters (Å^2^)

**Table:**

	*U*^11^	*U*^22^	*U*^33^	*U*^12^	*U*^13^	*U*^23^
S1	0.0215 (2)	0.0334 (3)	0.0307 (3)	0.0006 (2)	0.00107 (18)	−0.0006 (2)
O1	0.0239 (7)	0.0404 (9)	0.0390 (8)	0.0016 (6)	0.0082 (6)	−0.0030 (7)
O2	0.0295 (8)	0.0462 (10)	0.0373 (8)	−0.0022 (7)	−0.0046 (6)	−0.0019 (7)
O3	0.0269 (7)	0.0359 (8)	0.0334 (8)	−0.0061 (6)	−0.0021 (6)	0.0031 (6)
O4	0.0277 (7)	0.0370 (8)	0.0296 (7)	−0.0034 (6)	−0.0011 (6)	0.0033 (6)
N1	0.0228 (8)	0.0292 (9)	0.0267 (8)	−0.0014 (7)	0.0016 (6)	−0.0016 (7)
N2	0.0238 (8)	0.0316 (9)	0.0288 (8)	0.0000 (7)	0.0027 (6)	0.0034 (7)
C1	0.0293 (10)	0.0288 (11)	0.0283 (10)	0.0018 (8)	0.0052 (8)	0.0002 (8)
C2	0.0357 (11)	0.0340 (12)	0.0301 (10)	0.0059 (9)	0.0010 (8)	0.0011 (9)
C3	0.0528 (14)	0.0295 (11)	0.0295 (10)	0.0065 (10)	0.0088 (9)	0.0033 (9)
C4	0.0445 (12)	0.0261 (11)	0.0376 (11)	0.0015 (9)	0.0166 (9)	0.0016 (9)
C5	0.0310 (10)	0.0286 (10)	0.0349 (11)	0.0022 (8)	0.0093 (8)	−0.0004 (9)
C6	0.0260 (10)	0.0252 (10)	0.0287 (9)	0.0024 (8)	0.0061 (7)	−0.0014 (8)
C7	0.0205 (9)	0.0307 (10)	0.0275 (9)	0.0009 (7)	0.0040 (7)	−0.0032 (8)
C8	0.0219 (9)	0.0280 (10)	0.0260 (9)	0.0002 (7)	0.0025 (7)	−0.0001 (8)
C9	0.0239 (9)	0.0298 (10)	0.0288 (10)	0.0013 (8)	0.0062 (7)	−0.0004 (8)
C10	0.0436 (13)	0.0346 (12)	0.0344 (11)	0.0039 (10)	0.0025 (9)	−0.0071 (9)
C11	0.0256 (10)	0.0294 (10)	0.0353 (11)	0.0050 (8)	0.0117 (8)	0.0044 (9)
C12	0.0358 (11)	0.0349 (12)	0.0317 (10)	0.0027 (9)	0.0099 (8)	0.0020 (9)
C13	0.0440 (13)	0.0511 (15)	0.0342 (11)	0.0086 (11)	0.0118 (10)	0.0098 (11)
C14	0.0510 (15)	0.0430 (14)	0.0551 (15)	0.0076 (12)	0.0188 (12)	0.0207 (12)
C15	0.0505 (15)	0.0320 (13)	0.0688 (17)	−0.0029 (11)	0.0184 (13)	0.0108 (12)
C16	0.0370 (12)	0.0339 (12)	0.0468 (13)	−0.0009 (10)	0.0082 (10)	0.0036 (10)

### Geometric parameters (Å, º)

**Table:**

S1—O2	1.4228 (15)	C5—C6	1.390 (3)
S1—O1	1.4288 (15)	C5—H5	0.9500
S1—N1	1.6316 (18)	C6—C7	1.463 (3)
S1—C1	1.756 (2)	C7—C8	1.351 (3)
O3—C7	1.338 (2)	C8—C9	1.458 (3)
O3—H3O	0.8400	C10—H10A	0.9800
O4—C9	1.249 (2)	C10—H10B	0.9800
N1—C8	1.436 (2)	C10—H10C	0.9800
N1—C10	1.478 (3)	C11—C12	1.384 (3)
N2—C9	1.344 (3)	C11—C16	1.389 (3)
N2—C11	1.415 (3)	C12—C13	1.384 (3)
N2—H2N	0.8800	C12—H12	0.9500
C1—C2	1.377 (3)	C13—C14	1.372 (4)
C1—C6	1.405 (3)	C13—H13	0.9500
C2—C3	1.385 (3)	C14—C15	1.383 (4)
C2—H2	0.9500	C14—H14	0.9500
C3—C4	1.386 (3)	C15—C16	1.380 (3)
C3—H3	0.9500	C15—H15	0.9500
C4—C5	1.376 (3)	C16—H16	0.9500
C4—H4	0.9500		
			
O2—S1—O1	119.39 (9)	O3—C7—C6	114.78 (17)
O2—S1—N1	108.21 (10)	C8—C7—C6	122.84 (18)
O1—S1—N1	107.76 (9)	C7—C8—N1	120.90 (17)
O2—S1—C1	109.76 (10)	C7—C8—C9	121.19 (18)
O1—S1—C1	108.43 (10)	N1—C8—C9	117.89 (17)
N1—S1—C1	101.85 (9)	O4—C9—N2	123.74 (19)
C7—O3—H3O	109.5	O4—C9—C8	120.11 (18)
C8—N1—C10	115.09 (16)	N2—C9—C8	116.15 (17)
C8—N1—S1	113.31 (14)	N1—C10—H10A	109.5
C10—N1—S1	116.32 (13)	N1—C10—H10B	109.5
C9—N2—C11	128.34 (17)	H10A—C10—H10B	109.5
C9—N2—H2N	115.8	N1—C10—H10C	109.5
C11—N2—H2N	115.8	H10A—C10—H10C	109.5
C2—C1—C6	121.9 (2)	H10B—C10—H10C	109.5
C2—C1—S1	121.73 (16)	C12—C11—C16	120.2 (2)
C6—C1—S1	116.40 (15)	C12—C11—N2	122.6 (2)
C1—C2—C3	119.2 (2)	C16—C11—N2	117.26 (19)
C1—C2—H2	120.4	C11—C12—C13	119.1 (2)
C3—C2—H2	120.4	C11—C12—H12	120.4
C4—C3—C2	119.7 (2)	C13—C12—H12	120.4
C4—C3—H3	120.2	C14—C13—C12	121.1 (2)
C2—C3—H3	120.2	C14—C13—H13	119.4
C5—C4—C3	121.1 (2)	C12—C13—H13	119.4
C5—C4—H4	119.5	C13—C14—C15	119.5 (2)
C3—C4—H4	119.5	C13—C14—H14	120.2
C4—C5—C6	120.4 (2)	C15—C14—H14	120.2
C4—C5—H5	119.8	C16—C15—C14	120.4 (2)
C6—C5—H5	119.8	C16—C15—H15	119.8
C5—C6—C1	117.81 (18)	C14—C15—H15	119.8
C5—C6—C7	121.73 (18)	C15—C16—C11	119.7 (2)
C1—C6—C7	120.43 (18)	C15—C16—H16	120.2
O3—C7—C8	122.38 (18)	C11—C16—H16	120.2
			
O2—S1—N1—C8	169.66 (13)	C1—C6—C7—C8	17.5 (3)
O1—S1—N1—C8	−59.96 (15)	O3—C7—C8—N1	−178.49 (17)
C1—S1—N1—C8	54.02 (15)	C6—C7—C8—N1	1.4 (3)
O2—S1—N1—C10	32.92 (18)	O3—C7—C8—C9	0.1 (3)
O1—S1—N1—C10	163.30 (15)	C6—C7—C8—C9	179.90 (18)
C1—S1—N1—C10	−82.72 (16)	C10—N1—C8—C7	96.1 (2)
O2—S1—C1—C2	29.0 (2)	S1—N1—C8—C7	−41.2 (2)
O1—S1—C1—C2	−103.00 (19)	C10—N1—C8—C9	−82.5 (2)
N1—S1—C1—C2	143.52 (18)	S1—N1—C8—C9	140.21 (16)
O2—S1—C1—C6	−151.62 (16)	C11—N2—C9—O4	0.2 (3)
O1—S1—C1—C6	76.36 (17)	C11—N2—C9—C8	179.09 (18)
N1—S1—C1—C6	−37.12 (18)	C7—C8—C9—O4	−4.3 (3)
C6—C1—C2—C3	−0.7 (3)	N1—C8—C9—O4	174.32 (18)
S1—C1—C2—C3	178.60 (17)	C7—C8—C9—N2	176.80 (18)
C1—C2—C3—C4	−0.8 (3)	N1—C8—C9—N2	−4.6 (3)
C2—C3—C4—C5	0.8 (3)	C9—N2—C11—C12	23.9 (3)
C3—C4—C5—C6	0.8 (3)	C9—N2—C11—C16	−156.9 (2)
C4—C5—C6—C1	−2.2 (3)	C16—C11—C12—C13	0.6 (3)
C4—C5—C6—C7	175.80 (19)	N2—C11—C12—C13	179.77 (19)
C2—C1—C6—C5	2.2 (3)	C11—C12—C13—C14	−0.7 (3)
S1—C1—C6—C5	−177.17 (15)	C12—C13—C14—C15	0.3 (4)
C2—C1—C6—C7	−175.82 (19)	C13—C14—C15—C16	0.2 (4)
S1—C1—C6—C7	4.8 (3)	C14—C15—C16—C11	−0.2 (4)
C5—C6—C7—O3	19.4 (3)	C12—C11—C16—C15	−0.2 (3)
C1—C6—C7—O3	−162.66 (18)	N2—C11—C16—C15	−179.3 (2)
C5—C6—C7—C8	−160.5 (2)		

### Hydrogen-bond geometry (Å, º)

**Table:**

*D*—H···*A*	*D*—H	H···*A*	*D*···*A*	*D*—H···*A*
N2—H2*N*···O1^i^	0.88	2.26	2.987 (2)	140
C3—H3···O2^ii^	0.95	2.48	3.300 (3)	145
C13—H13···O4^iii^	0.95	2.48	3.339 (3)	151
N2—H2*N*···N1	0.88	2.27	2.724 (2)	112
O3—H3*O*···O4	0.84	1.79	2.534 (2)	146
C12—H12···O4	0.95	2.36	2.902 (3)	116

Symmetry codes: (i) −*x*, −*y*+1, −*z*; (ii) −*x*, *y*+1/2, −*z*−1/2; (iii) −*x*+1, *y*−1/2, −*z*+1/2.
